# The association between faecal host DNA or faecal calprotectin and feed efficiency in pigs fed yeast-enriched protein concentrate

**DOI:** 10.1017/S1751731119000818

**Published:** 2019-05-07

**Authors:** K. R. Slinger, A. H. Stewart, Z. C. T. R. Daniel, H. Hall, H. V. Masey O’Neill, M. R. Bedford, T. Parr, J. M. Brameld

**Affiliations:** 1 Nutritional Sciences, School of Biosciences, Sutton Bonington Campus, The University of Nottingham, Leicestershire LE12 5RD, United Kingdom; 2 Animal Production, Welfare and Veterinary Sciences, Harper Adams University, Newport, Shropshire TF10 8NB, United Kingdom; 3 AB Agri Limited, Innovation Way, Lynch Wood, Peterborough PE2 6FL, United Kingdom; 4 AB Vista, Woodstock Ct, Marlborough SN8 4AN, United Kingdom

**Keywords:** gut, swine, faeces, sloughing, *cytochrome b*

## Abstract

Gut cell losses contribute to overall feed efficiency due to the energy requirement for cell replenishment. Intestinal epithelial cells are sloughed into the intestinal lumen as digesta passes through the gastrointestinal tract, where cells are degraded by endonucleases. This leads to fragmented DNA being present in faeces, which may be an indicator of gut cell loss. Therefore, measuring host faecal DNA content could have potential as a non-invasive marker of gut cell loss and result in a novel technique for the assessment of how different feed ingredients impact upon gut health. Faecal calprotectin (**CALP**) is a marker of intestinal inflammation. This was a pilot study designed to test a methodology for extracting and quantifying DNA from pig faeces, and to assess whether any differences in host faecal DNA and CALP could be detected. An additional aim was to determine whether any differences in the above measures were related to the pig performance response to dietary yeast-enriched protein concentrate (**YPC**). Newly weaned (∼26.5 days of age) Large White × Landrace × Pietrain piglets (8.37 kg ±1.10, *n* = 180) were assigned to one of four treatment groups (nine replicates of five pigs), differing in dietary YPC content: 0% (control), 2.5%, 5% and 7.5% (w/w). Pooled faecal samples were collected on days 14 and 28 of the 36-day trial. Deoxyribonucleic acid was extracted and quantitative PCR was used to assess DNA composition. Pig genomic DNA was detected using primers specific for the pig *cytochrome b* (***CYTB***) gene, and bacterial DNA was detected using universal *16S* primers. A pig CALP ELISA was used to assess gut inflammation. Dietary YPC significantly reduced feed conversion ratio (**FCR**) from weaning to day 14 (*P*<0.001), but not from day 14 to day 28 (*P* = 0.220). Pig faecal *CYTB* DNA content was significantly (*P* = 0.008) reduced in YPC-treated pigs, with no effect of time, whereas total faecal bacterial DNA content was unaffected by diet or time (*P*>0.05). Faecal CALP levels were significantly higher at day 14 compared with day 28, but there was no effect of YPC inclusion and no relationship with FCR. In conclusion, YPC reduced faecal *CYTB* DNA content and this correlated positively with FCR, but was unrelated to gut inflammation, suggesting that it could be a non-invasive marker of gut cell loss. However, further validation experiments by an independent method are required to verify the origin of pig faecal *CYTB* DNA as being from sloughed intestinal epithelial cells.

## Implications

The host DNA in faeces presumably originates from gut cell losses. This development of a non-invasive assessment method is potentially a novel indirect measurement of gut cell losses, although further validation experiments are required. A yeast-inclusion diet significantly reduced pig DNA in faeces and therefore may reduce gut cell losses. Turnover of gut cells contributes to feed efficiency; therefore, assessment of host DNA in faeces could potentially, following further methodology development, form the basis of a procedure to indirectly assess gut health and feed conversion ratio.

## Introduction

Global food security is driving demand for increased feed efficiency in livestock production, resulting from an ever-growing human population (Gill, *et al.*, 2010). One important component of feed efficiency that is often ignored is gut turnover, which relates to the continual proliferation, differentiation, movement and subsequent sloughing of epithelial cells lining the gastrointestinal tract. The rate at which this occurs can be very high (Bregendahl *et al.*, 2004), requires both energy and nutrients for replenishment of cells (Baskin and Taegtmeyer, 2011), and therefore influences the feed efficiency. Consequently, accurate measurements of gut turnover are important when attempting to establish ways to improve feed efficiency. Currently all methods to measure gut turnover are invasive or expensive involving the use of stable isotope-labelled amino acids (Buttery, 1981) or deuterium-labelled water and expensive detection systems (Gasier *et al.*, 2010). Hence a simple non-invasive method of measuring gut cell losses could potentially be extremely useful to understand growth efficiency and diet suitability.

Epithelial cells lining the small intestine are rapidly turned over and lost into the small intestine lumen where they are degraded by endonucleases, leading to DNA debris being present in faeces (Creamer, *et al.*, 1961; Loretz, *et al.*, 2006). Most literatures regarding DNA extraction from faeces in humans and animals relate to (i) determination of the microbial population in the gut (Ariefdjohan *et al.*, 2010); (ii) use in human diagnostics of particular cancers (Roperch *et al.*, 2015); (iii) species confirmation in wildlife research (Ramón-Laca *et al.*, 2015); or (iv) use in forensics (Boonseub *et al.*, 2012). In all situations, the extraction of sufficient DNA of a suitable quality is the primary concern since faecal material contains inhibitors, such as bile salts, complex polysaccharides and haemoglobin degradation products (McOrist *et al.*, 2002), that can impair analysis methods (e.g. PCR). Previous studies have assessed human faecal DNA content as a marker of gut inflammation due to the expected increase in gut cell loss as a result of disturbance to the gut homeostasis (Vincent, *et al.*, 2015); however, to the best of our knowledge, the methodology has not been assessed in production animals. Our overall hypothesis is that the concentration of animal DNA present in faeces will be directly proportional to the number of cells lost from the gut. Therefore, the aim of this study was to use DNA extraction and PCR methodology to detect animal (host) DNA in faecal samples as a potential novel, simple, non-invasive measure of gut cell losses. However, faecal DNA will also include DNA from a range of other sources including bacteria and feed ingredients, meaning a host species-specific gene target is required. Mitochondrial DNA gene targets have previously been used as host-specific markers in studies assessing faecal contamination of water sources (Martellini *et al.*, 2005). Another advantage of using mitochondrial gene targets is the relatively high copy number in cells, including the sloughed epithelial cells present in faecal material (Caldwell, *et al.*, 2011). DNA extracted from faeces is likely to be highly degraded, so choosing a high copy number gene should increase the likelihood of detection. Pig *cytochrome b* (***CYTB***) was therefore chosen as the gene for assessing host DNA content in pig faeces in the present study.

This was a pilot study designed to explore whether host DNA content in faeces could be extracted and quantified and whether host DNA content in faeces was related to animal performance measures that might be influenced by gut cell sloughing. Inclusion of yeast extract in livestock diets had previously been shown to affect gut morphology, by increasing gut maturation, and also significantly improved animal performance (Muthusamy *et al.*, 2011). Therefore, inclusion of yeast-enriched protein concentrate (**YPC**) was used as a dietary treatment in this pilot study to test the hypothesis, since a significant reduction in gut cell sloughing was expected in YPC-treated animals, which should then be detected using the *CYTB* DNA assay.

Faecal calprotectin (**CALP**) levels have previously been used as a marker of gut inflammation in pigs (Lallès and Fagerhol, 2005). As previously mentioned, studies in humans have assessed human DNA content in faeces as a marker of bowel inflammation (Vincent, *et al.*, 2015). Since gut turnover might be influenced by gut inflammation, we also assessed the CALP levels in pig faeces along with any relationships between faecal CALP, *CYTB* DNA and animal performance, particularly feed conversion ratio (**FCR**).

## Material and methods

### Animals

The feeding study was carried out at Harper Adams University and was approved by the internal ethical review committee (Approval number: 00583940-STAFF-1-201607). The study did not use any ‘regulated procedures’ and was therefore not carried out under Home Office Licence. Newly weaned Large White × Landrace × Pietrain piglets (8.37 ± 1.10 kg, *n* = 180) were assigned to one of four dietary treatment groups (*n* = 9 pens per treatment, 5 animals per pen). The piglets were in single sex pens, with both males and females used in the study, but balanced across the treatment groups. There were five pens of males and four pens of females on each treatment. The pens were 1.5 m² with plastic slatted floors, in line with the commercial Assured Food Standard requirement of 0.3 m² per animal up to 30 kg in body weight. The diets differed in YPC (AB Agri) content, as follows: 0% (control), 2.5%, 5% and 7.5% (w/w) (Table 1). Diets were formulated by AB Agri Limited. All diets were formulated to be equivalent in net energy and standard ileal digestible lysine, methionine, threonine, tryptophan and valine and were balanced using potato protein and wheat. Diet 1 was offered from weaning (∼26.5 days) to day 14 of the trial, and diet 2 was offered from day 14 to day 28 (Table 1). No therapeutic zinc oxide or dietary antibiotics were used. Animals were weighed at the beginning of the trial (at weaning), on day 14 and day 28. Feed intake was determined by offering a specified weight of feed, measuring the amount of feed weighed back at the end of each week and using these values to calculate feed eaten. FCR was calculated as feed intake divided by animal body weight gain and provided as an average for each experimental pen. A single fresh faecal sample was taken immediately after defecation from a minimum of three pigs from each pen at weighing and these samples were then pooled for the pen. The pooled sample was approximately 40 g in weight. Weighing of the pigs was typically carried out at midday. The YPC product is a maize-based protein concentrate with a high content (30%) of inactive yeast from the bioethanol production industry. Its nutritional value is shown in Table 2.

**Table 1:** *Composition of pig diets*

% Ingredient unless otherwise specified	Diet 1 (wean to day 14)				Diet 2 (day 14 to day 28)			
	Control 0% YPC	2.5% YPC	5.0% YPC	7.5% YPC	Control 0% YPC	2.5% YPC	5.0% YPC	7.5% YPC
Raw wheat	38.3	37.4	35.6	33.9	53.8	52.0	49.8	47.6
Micronised barley	15.0	15.0	15.0	15.0	15.0	15.0	15.0	15.0
Micronised oats	5.0	5.0	5.0	5.0	0.0	0.0	0.0	0.0
HiPro soya	10.0	10.0	10.0	10.0	15.0	15.0	15.0	15.0
Soya oil	5.1	5.3	5.5	5.7	1.1	1.3	1.6	1.8
YPC	0.0	2.5	5.0	7.5	0.0	2.5	5.0	7.5
AlphaSoy 530	7.5	7.5	7.5	7.5	5	5	5	5
Vitamin and mineral premix	0.5	0.5	0.5	0.5	0.5	0.5	0.5	0.5
Whey powder	10.4	10.4	10.4	10.4	3.5	3.5	3.5	3.5
Potato protein	4.0	2.0	1.0	0.0	2.0	1.0	0.5	0.0
L-Lysine HCl	0.57	0.66	0.68	0.70	0.59	0.61	0.60	0.59
DL-Methionine	0.27	0.29	0.28	0.28	0.26	0.26	0.25	0.24
L-Threonine	0.27	0.31	0.31	0.31	0.28	0.28	0.26	0.25
L-Tryptophan	0.06	0.07	0.07	0.08	0.05	0.05	0.05	0.05
L-Valine	0.15	0.20	0.21	0.22	0.14	0.15	0.14	0.13
Limestone flour	0.32	0.38	0.44	0.50	0.08	0.14	0.19	0.25
Dicalcium phosphate	1.87	1.86	1.84	1.82	2.04	2.02	2.00	1.98
Salt	0.00	0.00	0.00	0.00	0.04	0.03	0.04	0.04
Sodium biocarbonate	0.58	0.61	0.60	0.60	0.62	0.61	0.58	0.55
Calculated nutritive values								
Protein	19.7	19.5	19.9	20.2	19.3	19.7	20.3	20.9
Digestible energy (MJ/kg)	15.3	15.2	15.2	15.2	14.1	14.2	14.2	14.2
Oil	7.6	7.7	7.9	8.2	3.3	3.6	3.8	4.1
Fibre	2.2	2.3	2.4	2.5	2.4	2.5	2.6	2.7
Calcium	0.77	0.79	0.81	0.83	0.77	0.79	0.80	0.82
Digestible phosphorus	0.4	0.4	0.4	0.4	0.4	0.4	0.4	0.4
Lysine^a^	1.35	1.35	1.35	1.35	1.28	1.28	1.28	1.28
Methionine^a^	0.54	0.54	0.54	0.54	0.51	0.51	0.51	0.51
Methionine + Cysteine^a^	0.81	0.81	0.81	0.82	0.78	0.79	0.79	0.80
Threonine^a^	0.90	0.90	0.90	0.90	0.86	0.86	0.86	0.86
Isoleucine^a^	0.72	0.68	0.67	0.66	0.69	0.67	0.68	0.69
Tyrosine^a^	0.57	0.53	0.52	0.52	0.54	0.54	0.55	0.57
Valine^a^	0.95	0.95	0.95	0.95	0.90	0.90	0.90	0.90

YPC=yeast-enriched protein concentrate.

a Amino acid levels are expressed as standardised ileal digestible content.

**Table 2:** *Nutrient composition of YPC fed to the pigs*

Ingredient	YPC
CP (%)	49.0
DM (%)	95.0
Gross energy (MJ/kg)	20.2
Ether extract (%)	3.80
Crude fibre (%)	4.50
Ash (%)	5.0
Calcium (%)	0.02
Phosphorus (%)	0.92
NDF	2.9
ADF	8.5
Starch	3.0
Sugars	0.5

YPC = yeast-enriched protein concentrate.

### DNA extraction

Genomic DNA was extracted from pig faeces using a modified phenol chloroform extraction method, followed by ethanol precipitation. All centrifugation steps were at 16 000**×g** and room temperature. Faeces were not dried prior to weighing. Briefly, 30 mg of frozen faeces was homogenised in 600 µl Nuclei Lysis Solution (Promega) using a hand-held homogeniser, the solution centrifuged for 2 min and the resulting supernatant used for a standard phenol : chloroform : isoamyl alcohol (25 : 24 : 1) (Sigma) extraction followed by an overnight precipitation with 3 M sodium acetate (0.1 × sample volume) and 100% (v/v) ethanol (2.5 × (sample + sodium acetate)). The DNA was re-suspended in 100 µl of water (DNase and RNase free). Following re-suspension, 25 µl of DNA was incubated with 0.5 µl of RNase A (Promega) at 37°C for 15 min to remove RNA contaminants. After incubation, 200 µl of water (DNase and RNase free) was added to increase the volume and a further two-step phenol : chloroform : isoamyl alcohol (25 : 24 : 1) (Sigma) clean-up was carried out with a final overnight precipitation with 3 M sodium acetate (0.1 × sample volume) and 100% (v/v) ethanol (2.5 × (sample + sodium acetate)). Full method is presented in Supplementary Material S1. The DNA was re-suspended in a final volume of 10 µl. Deoxyribonucleic acid concentration (ng/µl) and DNA purity (260 nm/280 nm) were measured using the NanoDrop™ 2000 (Thermo Scientific) spectrophotometer. DNA yield was calculated as nanogram per milligram of faecal material. Extracted DNA samples were stored at –20˚C prior to analysis.

### Quantitative PCR

To assess the DNA composition, SYBR Green (Roche) quantitative PCR methodology was used. Primers for detecting host (pig) DNA were designed specifically to the pig *CYTB* gene (Accession no. AY920909.1) [forward primer 5′-TTCATAGGCTACGTCCTGCC-3′ and reverse primer 5′-TCGTGTGAGGGTTGCTTTGT-3′], with a predicted amplicon length of 150 bp. For the detection of bacterial *16S* DNA, published universal *16S* primers (Mieszkin *et al.*, 2009) were used with a predicted amplicon length of 142 bp. Polymerase chain reaction was performed on 384-well plates using a LightCycler® 480 (Roche) instrument. Each well contained 7.5 µl of SYBR Green (Roche) Mastermix, 1.6 µl of water (DNase and RNase free), 0.45 µl of each primer (10 µM, Forward and Reverse), and 5 µl of either the PCR standard, sample, or water (DNase and RNase free) as a negative control. The PCR protocol used was as follows: pre-incubation at 95˚C for 5 min, followed by 45 amplification cycles (95˚C for 10 s, 60˚C for 15 s, 72˚C for 15 s), a single melt cycle (95˚C for 5 s followed by 65˚C for 1 min) and then a cooling cycle of 40˚C for 10 s. A melt cycle was carried out to confirm a single product was produced in the PCR reaction. A pool of extracted faecal DNA samples diluted to 10 ng/µl was used in a fourfold dilution series to create standard curves for both *CYTB* and bacterial *16S* DNA. Individual pig faecal DNA samples were then diluted 1 in 8 and tested in triplicate against the standard curve. Negative template controls (DNase and RNase free water) were also tested in triplicate at two points on the plate. Intra-assay CV for *CYTB* DNA assay, which takes into account the DNA extraction, is 0.66%. Intra-assay CV for the bacterial *16S* DNA assay is 0.93%. The assays are carried out on the same day with all samples analysed on the same plate and therefore there is no reported inter-assay CV. Polymerase chain reaction products were verified by Sanger Sequencing (BioScience, Nottingham, UK).

### Pig Calprotectin ELISA

Calprotectin content in faeces was assessed using the Pig Calprotectin (**CALP**) ELISA kit (Cusabio, China) according to manufacturer’s instructions, using a FLUOstar® Omega platereader (BMG LABTECH Ltd, UK) to measure absorbance at 450 nm. Intra-assay CV is <8% and inter-assay CV is <10%. The protocol was optimised for use with faecal samples, whereby samples were diluted 1 : 5 with sample diluent rather than the suggested 1 : 500 for plasma or serum samples. Pig faecal samples were tested in duplicate, requiring two plates, and each plate contained a standard curve also in duplicate. The standard curve was created by generating a four-parameter logistic (4-PL) curve-fit using online software (elisaanalysis.com). The standard curve was then used to calculate CALP concentration values in the faecal samples and the resulting value was multiplied by the dilution factor (x5) to give a final faecal CALP concentration, as per the manufacturer’s instructions.

### Statistical analysis of results

All data were analysed using *Genstat Statistical Software* (17^th^ edition), with significance at *P*<0.05. One-way ANOVA was used to assess the effects of diet on animal performance (BW, daily feed intake, daily live weight gain and FCR). Two-way (factors: diet × day of trial, blocking for pen) ANOVA were used to assess whether there were any effects on the resulting DNA concentration (ng/µl), DNA quality (260 nm/280 nm), DNA yield (ng/mg faecal material) and DNA contents (*CYTB*, *16S* and *CYTB* : *16S* ratio). Three-way ANOVA (factors: diet × day of trial × plate, blocking for pen) was used to analyse the faecal pig CALP levels. Where significant differences were observed, post hoc Bonferroni tests were then performed. The relationships between pig faecal *CYTB* DNA content and FCR and pig faecal CALP and FCR were assessed by Pearson’s correlation for both time points separately.

## Results

### Effects of Yeast Protein Concentrate on pig performance

There were no observable health issues to report and there were no significant treatment differences in daily intake or growth rate (Table 3). Pigs fed diets containing 2.5%, 5% or 7.5% YPC had significantly reduced FCR recorded from wean to day 14 (*P*<0.001) and over the entire 28-day trial (wean-day 28, *P* = 0.019) for 5% and 7.5% YPC inclusion compared to animals on the control diet (Table 3). However, there was no effect (*P* = 0.220) of YPC on FCR from day 14 to day 28 (Table 3).

**Table 3:** *The effect of dietary YPC on pig performance*

Animal performance	YPC inclusion level (%)				SED	*P*-value
	0	2.5	5.0	7.0		
Average BW at weaning (kg)	8.38	8.33	8.38	8.37	0.025	0.175
Average BW at day 14 (kg)	12.30	12.91	13.03	12.79	0.291	0.081
Average BW at day 28 (kg)	21.95	21.66	22.68	21.84	0.666	0.457
DFI wean to day 14 (g/pig per day) (diet 1)	340	339	338	317	19.0	0.561
DFI day 14 to day 28 (g/pig per day) (diet 2)	830	776	823	791	41.0	0.542
DFI wean to day 28 (g/pig per day) (treatment diets)	580	557	580	554	27.0	0.585
DLWG wean to day 14 (g/pig per day) (diet 1)	280	327	332	316	21.1	0.076
DLWG day 14 to day 28 (g/pig per day) (diet 2)	687	625	689	646	36.4	0.240
DLWG wean to day 28 (g/pig per day) (treatment diets)	483	476	511	481	24.6	0.510
FCR wean to day 14 (diet 1)	1.24^a^	1.04^b^	1.02^b^	1.01^b^	0.048	<0.001
FCR day 14 to day 28 (diet 2)	1.21	1.25	1.20	1.23	0.025	0.220
FCR wean to day 28 (treatment diets)	1.21^a^	1.17^ab^	1.14^b^	1.15^b^	0.023	0.019

DFI = daily feed intake; DLWG = daily live weight gain; FCR = feed conversion ratio (feed intake/weight gain); YPC = yeast-enriched protein concentrate; SED = standard error of the differences of the means.

Means with different superscript letters in the same row differ significantly.

### DNA extraction

**Figure 1:**
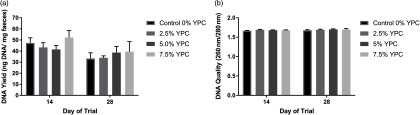
The effect of an YPC-inclusion diet and when the pig faeces were collected on (a) DNA yield (ng/mg faecal material) and (b) the DNA quality (260 nm/280 nm). Mean values + Standard Error of the Mean, *n* = 9 for all groups. SEDs for YPC × day interaction=: (a) 7.60 and (b) 0.021. DNA yield was greater at day 14 compared with day 28 (*P* = 0.016). YPC=yeast-enriched protein concentrate; SED=standard error of the differences of the means.

DNA yield (ng/mg faecal material) was significantly (*P* = 0.016) higher at day 14 compared with day 28 (Figure 1a). However, there was no effect of dietary treatment (*P* = 0.551), nor any interaction (*P* = 0.739) between day and dietary treatment (Figure 1a). When analysing DNA quality (260 nm/280 nm) there were no significant (*P*>0.05) interactions or effects of either diet or day of the trial (Figure 1b).

### Effects of Yeast Protein Concentrate on faecal host and bacterial DNA composition and calprotectin concentration

**Figure 2:**
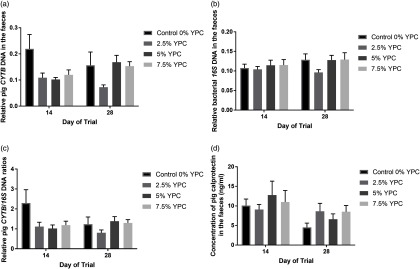
The effect of an YPC-inclusion diet and when the faeces were collected on faecal (a) pig CYTB DNA content, (b) bacterial *16S* DNA content, (c) pig CYTB DNA normalised to bacterial *16S* DNA content and (d) pig CALP concentrations (ng/ml). Mean values + SEM, *n* = 9 for all groups. SEDs for YPC × day interaction=: (a) 0.045, (b) 0.018, (c) 0.445 and (d) 0.600. There were significant effects of diet on both CYTB DNA content (*P* = 0.008) and CYTB normalised to *16S* DNA (*P* = 0.045), but not *16S* DNA content and no significant diet × day interactions or effects of day (*P*>0.05). See Table 4 for post hoc Bonferroni analyses for effects of diet on CYTB DNA content and CYTB normalised to *16S* DNA. Pig CALP concentration was greater at day 14 compared with day 28 (*P* = 0.009). YPC=yeast-enriched protein concentrate; CYTB=*cytochrome b*; SED=standard error of the differences of the means; CALP=calprotectin.

There was no day × YPC interaction (*P* = 0.222) nor any effect of day (*P* = 0.997) on pig *CYTB* DNA in the faeces, but there was a significant (*P* = 0.008) effect of YPC diet (Figure 2a). Bonferroni post hoc analysis showed that faeces from animals on the control diet (0% YPC) contained significantly (*P*<0.01) more pig *CYTB* DNA than those on the 2.5% YPC diet (Figure 2a and Table 4). Interestingly, even though there was no day × YPC interaction, all three YPC diets appeared to reduce faecal pig *CYTB* DNA contents at day 14, but only 2.5% YPC did so at day 28 (Figure 2a).

**Table 4:** *The effect of diet on mean faecal host DNA content in pig faecal samples collected on days 14 and 28 combined*

Faecal DNA component	Mean faecal DNA content in dietary group				SED	*P*-value for effect of diet (ANOVA)
	Control (0% YPC)	2.5% YPC	5% YPC	7.5% YPC		
*CYTB* DNA	0.188^A^	0.091^B^	0.135^AB^	0.136^AB^	0.0259	0.008
*CYTB* DNA normalised for *16S* DNA	1.77^a^	0.96^b^	1.20^ab^	1.24^ab^	0.278	0.045

CYTB=cytochrome b; YPC=yeast-enriched protein concentrate; SED=standard error of the differences of the means.

a,b Samples differing in lowercase letters indicate significant statistical differences at *P*<0.05.

A,B Samples differing in uppercase letters indicate significant statistical differences at *P*<0.01.

In contrast, there were no significant effects of diet (*P* = 0.348), day (*P* = 0.205) nor an interaction (*P* = 0.602) on bacterial *16S* DNA content in the faeces (Figure 2b). As there were no differences in bacterial *16S* DNA, bacterial DNA was used to normalise the pig *CYTB* DNA content (Figure 2c). There was still a significant (*P* = 0.045) effect of YPC diet observed when *CYTB* DNA was normalised for bacterial *16S* DNA content, with animals on the control diet (0% YPC) having significantly (*P*<0.05) more *CYTB/16S* DNA in their faeces compared to those on the 2.5% YPC diet (Figure 2c and Table 4), with no effect of day (*P* = 0.368) nor a day × diet interaction (*P* = 0.206).

Pig faecal CALP is a marker of inflammation and the levels were significantly higher at day 14 compared to day 28 (*P* = 0.009), indicating more intestinal inflammation closer to weaning (Figure 2d). However, there was no significant (*P* = 0.709) effect of diet nor a day × diet interaction (*P* = 0.391).

### Relationship between host DNA, calprotectin and feed conversion ratio

**Figure 3:**
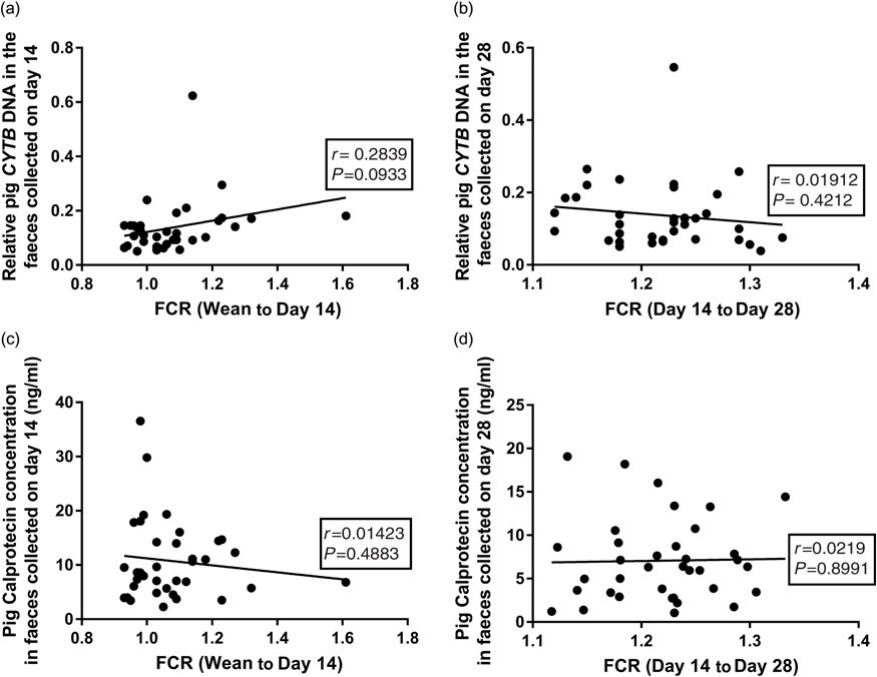
(a) The relationship between the pig *CYTB* DNA content in faeces at day 14 and recorded FCR (weaning to day 14). (b) The relationship between the pig *CYTB* DNA content in faeces at day 28 with FCR (day 14 to day 28). (c) The relationship between pig faecal CALP concentration (ng/ml) at day 14 and FCR (weaning to day 14). (d) The relationship between pig faecal CALP (ng/ml) at day 28 and FCR (day 14 to day 28). CYTB=*cytochrome b*; CALP=calprotectin; FCR=feed conversion ratio.

Pearson correlations were used to investigate relationships between the faecal DNA or CALP contents and feed efficiency. There was a trend for a correlation (*r* = 0.2839, *P* = 0.093) between the faecal pig *CYTB* DNA content (at day 14) and the recorded FCR (wean to day 14) over the early stages, where a significant effect of diet on FCR was observed (Figure 3a and Table 3). However, there was no correlation (*P* = 0.393) at the later phase (day 14 to day 28; Figure 3b), where no effect of diet on FCR was observed (Table 3). Likewise, there were no significant correlations between faecal CALP concentrations and recorded FCR at either day 14 (Figure 3c, *P* = 0.4883) or day 28 (Figure 3d, *P* = 0.9667).

## Discussion

To the best of our knowledge, this is the first (pilot) study to measure host DNA in faeces as a non-invasive marker of gut cell losses, which contribute to feed efficiency in production animals. A yeast-inclusion diet was used to test the hypothesis as a previous study had shown that dietary yeast influenced gut morphology and animal performance (Muthusamy *et al.*, 2011). The current study shows that host *CYTB* DNA content in faeces may be a novel, simple, non-invasive marker for assessing gut cell losses. DNA yield was significantly greater in samples extracted from day 14 compared to samples extracted on day 28. The reason for this is unclear but it may be due to there being a greater level of feed digestion at day 28 as there being a more mature gut. Faecal DM was not measured and while it is unlikely to impact upon PCR, as measurements are performed on a constant DNA basis, this may account for the difference in DNA yield, although this is purely speculation. The DNA quality was unaffected by either diet or day of the trial the samples were collected. Polymerase chain reaction of both pig *CYTB* DNA and bacterial *16S* DNA was successful, as verified by Sanger Sequencing, illustrating that a modified phenol–chloroform extraction method was suitable for extracting sufficient DNA of a suitable quality from pig faeces. In order for a PCR-based methodology to be used for the evaluation of gut cell losses in faeces, the host gene target needs to be present in sufficient quantities in the faeces to be measurable, as faeces are a heterogeneous material. As pig *CYTB* was successfully amplified and measured using quantitative PCR, it appears to be suitable as a gene target for the assessment of host DNA in faeces.

The average growth performance of the pigs was as expected for the unit and no undesirable side effects of the treatment were noted. The YPC product contains a maize-derived protein, which is expected to provide a valuable source of amino acids within the diet. However, the product also contains around 30% inactive yeast, from the bioethanol process. This yeast component is expected to provide a functional benefit beyond the protein value. Halder *et al.* (2011) investigated a low level of inclusion of a similar product in the diets of heat-stressed broilers and found a significant improvement in feed efficiency. Yeast contains substantial amounts of mannan and glucan in the cell wall. These components have been suggested to exert a ‘pre-biotic-like’ mechanism, with varied performance-enhancing effects such as inhibition of pathogenic invasion, modulation of the host immune response and enhancing the host gut morphology, thereby limiting the number of pathogens in the gut lumen (Roto *et al.*, 2015). The main mechanism by which this occurs is thought to involve the mannan and glucan from the yeast cell wall binding to pathogenic bacteria, preventing their attachment to the intestinal epithelia and therefore enabling the host to reserve energy for growth, rather than repair and replenishment of epithelial cells (Roto *et al.*, 2015).

Pig *CYTB* DNA was significantly higher in faeces collected from the pigs on the control diet compared with pigs on the 2.5% YPC-inclusion diet throughout the study, although at day 14, animals on the 5% and 7.5% inclusion level also appeared to have less pig *CYTB* DNA in their faeces compared to the controls. The reason for this is uncertain but it could be due to gut maturation or microbiome development and the 2.5% YPC inclusion level being optimal for the gut health benefit via a ‘pre-biotic-like’ mechanism previously discussed. Conversely, bacterial *16S* DNA content was unaffected by either the diet or the age of the pigs. The fact that the bacterial *16S* DNA content remains unchanged suggests it could potentially be used as a normalising control for the analysis of host DNA content in livestock faeces. The level of host DNA in faeces was hypothesised to be directly proportional to the amount of gut cell sloughing. The results of this study indicate that there is significantly more gut cell sloughing in the control fed pigs than the animals on a yeast-inclusion diet. It is therefore possible that the yeast component exerted a protective effect on the gut epithelium as previously described. As these animals are newly weaned pigs, the results suggest that inclusion of YPC in the diet helps the animal cope better with the problems observed when animals are transitioning to solid feed (Fairbrother *et al.*, 2005).

Gut cell losses contribute to overall feed efficiency of the animal due to the high nutrient demand for replenishing the cells lining the small intestine (Bregendahl *et al.*, 2004). There was a trend for a relationship between FCR measured over the first 14 days of the trial and the pig *CYTB* DNA content in the faeces at day 14, but not at the later phase (day 14 to day 28). In the first 14 days of the study, significant differences in FCR between dietary treatments were observed, and equally no significant differences in FCR were observed at the later phase. These results suggest that the levels of host DNA content in faeces may be a useful method for assessing gut cell losses and their impact on feed efficiency. However, this relies on the assumption that host DNA in faeces originates only from cell sloughing in the gastrointestinal tract, which is yet to be confirmed.

Faecal CALP levels are a recognised measure of intestinal inflammation and have been widely studied in humans (Fagerberg *et al.*, 2005). However, fewer studies have been carried out in animals. Lallès and Fagerhol (2005) determined reference values for faecal CALP levels in pigs of different ages and lactation stage but the present study has assessed dietary intervention of newly weaned piglets. Pig faecal CALP was significantly higher at day 14 of the trial compared with day 28, with no influence of the YPC diet. Previous studies have shown that dietary inclusion of chitosan can reduce faecal CALP and therefore intestinal inflammation in pigs (Xiao, *et al.*, 2014), but the YPC had no effect in the present study. The only differences in pig faecal CALP concentration (ng/ml) were caused by the age of the animal (day 14 > day 28), whereas differences in pig *CYTB* DNA were caused by diet. This shows that the pig *CYTB* DNA content is unrelated to the levels of CALP in the faeces, which is a marker of inflammation. Conversely, Varela *et al.* (2009) found a significant, but weak, correlation between human faecal DNA content and CALP content and hypothesised the weak relationship was due to the different origins of the markers.

In conclusion, dietary YPC positively influenced FCR in newly weaned piglets and reduced faecal *CYTB* DNA content, suggesting that faecal *CYTB* DNA content may be a novel non-invasive measure of gut cell losses. However, further work is required to elucidate the origin of the faecal host DNA and determine how sensitive the faecal DNA assay is. These experiments will form the basis for validation studies where the DNA assay will be compared with established methodologies.

## Notes

K. R. Slinger, 0000-0002-7417-165X, A. H. Stewart. https://orcid.org/0000-0003-0309-5343, T. Parr, 0000-0003-2490-9457, J. M. Brameld, 0000-0003-4942-080X

## Software and data repository resources

None of the data were deposited in an official repository.

## Supplementary material

For supplementary material accompanying this paper visit https://doi.org/10.1017/S1751731119000818. click here to view supplementary material
