# Supplementary material for: The association between faecal host DNA or faecal calprotectin and feed efficiency in pigs fed yeast-enriched protein concentrate
Source: Animal. 2019 May 7;13(11):2483–91. doi: 10.1017/S1751731119000818 (PMC6801643; doi:10.1017/S1751731119000818)
Supplement: Supplementary file 1 [file S1751731119000818sup001.docx]

***animal* journal**

**The association between faecal host DNA or faecal calprotectin and feed efficiency in pigs fed yeast enriched protein concentrate**

K.R. Slinger, A.H. Stewart, Z.C.T.R Daniel, H. Hall, H.V. Masey O’Neill, M.R. Bedford, T. Parr, J.M. Brameld

**Supplementary Material S1**: DNA extraction method

Genomic DNA was extracted from pig faeces using a modified phenol chloroform extraction method, followed by ethanol precipitation. All centrifugation steps were at 16,000xg and at room temperature. A detailed extraction protocol was as follows:

1. Weigh out 30mg of faeces into a 1.5ml tube
2. Add 600µl of Nuclei Lysis Solution (Product number: A7941, Promega UK, Southampton, UK) to the tube containing 30mg of faeces
3. Homogenise using a handheld homogeniser
4. Centrifuge sample for 2 minutes
5. Retain supernatant and discard pellet
6. Add an equal volume of phenol:chloroform:isoamyl alcohol (25:24:1) (Product number: P3803, Sigma-Aldrich Company Ltd, Dorset, UK) to the supernatant and vortex for 10 seconds followed by gentle mixing for 1 minute
7. Centrifuge sample for 2 minutes
8. Remove upper aqueous phase and transfer to a clean 1.5ml tube
9. Repeat steps 6-8 (taking into account the new starting volume)
10. To the final volume of aqueous solution add (0.1 x volume of sample) of 3M sodium acetate (NaAc) pH 5.2 and (2.5 x (volume of sample + NaAc)) of 100% (v/v) ethanol
11. Incubate at -20˚C overnight
12. Following overnight incubation, centrifuge for 15 minutes
13. Discard supernatant and retain pellet
14. To the pellet, add 500µl of 70% (v/v) ethanol to wash the DNA pellet
15. Centrifuge sample for 2 minutes
16. Discard supernatant and air dry the pellet at room temperature for 10 minutes
17. Re-suspend the pellet in 100µl of water (DNase & RNase free) and vortex for 10 seconds to ensure the DNA is re-suspended
18. To remove RNA contamination, incubate 25µl of DNA with 0.5µl of RNase A (Product number: A7973, Promega UK, Southampton, UK) at 37˚C for 15 minutes
19. After incubation, add 200µl of water (DNase & RNase free) to the sample to increase the volume
20. Add 225µl of phenol:chloroform:isoamyl alcohol (25:24:1) (Sigma), vortex for 10 seconds and mix by gentle inversion for 1 minute
21. Centrifuge sample for 2 minutes
22. Remove upper aqueous phase and transfer to a clean 1.5ml tube
23. To the new volume of supernatant add another equal volume of phenol:chloroform:isoamyl alcohol (25:24:1) (Sigma), vortex for 10 seconds and mix by gentle inversion for 1 minute
24. Repeat steps 21-22
25. To the final volume of aqueous solution add (0.1 x volume of sample) of 3M sodium acetate (NaAc) pH 5.2 and (2.5 x (volume of sample + NaAc)) of 100% (v/v) ethanol
26. Incubate at -20˚C overnight
27. Following overnight incubation, centrifuge for 15 minutes
28. Discard supernatant and retain pellet
29. To the pellet, add 200µl of 70% (v/v) ethanol to wash the DNA pellet
30. Centrifuge sample for 2 minutes
31. Discard supernatant and air dry the pellet at room temperature for 10 minutes
32. Re-suspend the pellet in 10µl of water (DNase & RNase free) and vortex for 10 seconds to ensure the DNA is re-suspended
